# Contrast-Enhanced MicroCT for Virtual 3D Anatomical Pathology of Biological Tissues: A Literature Review

**DOI:** 10.1155/2019/8617406

**Published:** 2019-02-28

**Authors:** Sébastien de Bournonville, Sarah Vangrunderbeeck, Greet Kerckhofs

**Affiliations:** ^1^Prometheus, Division of Skeletal Tissue Engineering, KU Leuven, Leuven, Belgium; ^2^Biomechanics Section, Department of Mechanical Engineering, KU Leuven, Leuven, Belgium; ^3^Molecular Design and Synthesis, Department of Chemistry, KU Leuven, Leuven, Belgium; ^4^Biomechanics Lab, Institute of Mechanics, Materials and Civil Engineering, UCLouvain, Louvain-la-Neuve, Belgium; ^5^Department Materials Engineering, KU Leuven, Leuven, Belgium

## Abstract

To date, the combination of histological sectioning, staining, and microscopic assessment of the 2D sections is still the golden standard for structural and compositional analysis of biological tissues. X-ray microfocus computed tomography (microCT) is an emerging 3D imaging technique with high potential for 3D structural analysis of biological tissues with a complex and heterogeneous 3D structure, such as the trabecular bone. However, its use has been mostly limited to mineralized tissues because of the inherently low X-ray absorption of soft tissues. To achieve sufficient X-ray attenuation, chemical compounds containing high atomic number elements that bind to soft tissues have been recently adopted as contrast agents (CAs) for contrast-enhanced microCT (CE-CT); this novel technique is very promising for quantitative “virtual” 3D anatomical pathology of both mineralized and soft biological tissues. In this paper, we provided a review of the advances in CE-CT since the very first reports on the technology to date. Perfusion CAs for *in vivo* imaging have not been discussed, as the focus of this review was on CAs that bind to the tissue of interest and that are, thus, used for *ex vivo* imaging of biological tissues. As CE-CT has mostly been applied for the characterization of musculoskeletal tissues, we have put specific emphasis on these tissues. Advantages and limitations of multiple CAs for different musculoskeletal tissues have been highlighted, and their reproducibility has been discussed. Additionally, the advantages of the “full” 3D CE-CT information have been pinpointed, and its importance for more detailed structural, spatial, and functional characterization of the tissues of interest has been shown. Finally, the remaining challenges that are still hampering a broader adoption of CE-CT have been highlighted, and suggestions have been made to move the field of CE-CT imaging one step further towards a standard accepted tool for quantitative virtual 3D anatomical pathology.

## 1. Introduction

Since decades, histological slicing is the gold standard in the field of biomedical sciences for the evaluation and characterization of biological tissues. The broad range of class-specific (DNA, proteins, lipids, or carbohydrates) dyes that exist in the field allowed histology to become a highly versatile screening technique. Out of a single sample, histology can furnish a significantly high amount of (mostly) qualitative and quantitative characteristics of different kind of tissues [1]. Although having a highly discriminative power on both the tissue and cellular level, histological assessment (slicing, staining, imaging, and analysis) remains a challenging, time-consuming, and costly technique [2]. If the tissue is mineralized, decalcification might be required before slicing (which can take more than 1 week depending on the sample size) and, depending on the dimensions and heterogeneity of the tissue, a large number of slices might be required for a sufficient spatial analysis of the sample. Also, slicing the sample can only be performed under a restricted sectioning orientation and is subject to processing glitches and distortions. After staining the slices, the sections are typically assessed under a microscope. This results in a stack of two-dimensional (2D) microscope images, representing the three-dimensional (3D) arrangement of the sample. While the individual slices can be of very high quality (highly selective stains, high in-plane spatial, and contrast resolution), the resolution along the third dimension in space is often limited due to the restricted amount of sections that can be realistically sliced and imaged for one sample [3]. These drawbacks play an important role for the spatial assessment of highly heterogeneous tissues (e.g., trabecular bone and vascular networks), as well as to quantify the spatial inter-relationship between different tissues within one sample. For heterogeneous tissues, an ideal alternative 3D visualization technique should allow to (a) provide full 3D information and (b) provide sufficient spatial and contrast resolution for the structures of interest, while (c) allowing a broad field of view along any direction of the volume and (d) reducing the workload for sample preparation. Additionally, it should (e) increase the throughput, (f) provide a wide range of specific stains with sufficient contrast, and (g) limit the destructiveness of the methodology to the samples, as not to harm the tissue integrity nor hamper the complementarity with histology or other biochemical assays.

X-ray microfocus computed tomography (microCT) has proven to be a very powerful tool for 3D imaging of mineralized samples, in many biomedical fields [4]. Thanks to the penetrating power of X-rays, microCT provides a 3D density map of the samples (a). High-resolution microCT scans are achievable (<1 *µ*m voxel size) (b) and a high field of view to voxel size ratio can be obtained nowadays (c). Additionally, the workload for sample preparation before imaging is substantially lower compared to other imaging techniques (e.g., histology), and fast image acquisition and volume reconstruction times are feasible by means of the current high-resolution microCT devices, justifying the higher throughput potential of this technology (d, e). While one of the biggest advantages of microCT is its nondestructive character (no need for cutting samples) (g), a considerable limitation of this technology is its lack of specificity for different soft tissue types. Indeed, X-ray imaging techniques are suitable to investigate strongly absorbing samples, such as mineralized tissues, but it struggles with discriminating nonmineralized or soft biological tissues that have similar and weak X-ray absorbing properties. Consequently, it can be challenging to differentiate between soft tissues on X-ray radiographs and reconstructed microCT images.

The introduction of radio-opaque contrast agents (CA) enabled high-resolution microCT to become a very important tool in biomedical imaging because of their ability to bind to tissues of interest or to perfuse through them, hence increasing the X-ray attenuation coefficient of the tissues of interest. As reviewed further in this article, different CAs have been recently reported for contrast-enhanced microCT (CE-CT) of specific soft tissues (f). However, care should be taken that both the CA staining protocol and the image acquisition setup do not hamper the nondestructive character of the methodology. Hence, its complementarity with subsequent biochemical assays, while maintaining good image quality and specific imaging of the structures of interest, should be perceived, and it should not have any effect on the tissue integrity. In this paper, we review the recent advances in CE-CT since the very first reports on the technology.

It is important to note that this review will focus on single energy, polychromatic absorption CE-CT imaging, whereby the soft tissue contrast is generated by the CAs. We would briefly like to highlight, though, that other alternative X-ray-based imaging techniques also exist with which additional contrast can be introduced. Dual energy X-ray scanning, on the one hand, could be used to distinguish two compounds with a similar attenuation, but with a different k-edge (i.e., inner electron shell binding energy). By scanning at two different energies (before and after the specific k-edge), contrast enhancement of specific CAs can be achieved [5]. Phase contrast X-ray CT, on the other hand, provides information concerning changes in the phase of an X-ray beam that passes through an object. It can be used to, for example, enhance edges, which allows a better visualization of soft tissues [6, 7]. Polarization of the emitted light allows to focus it on a specific orientation, which could be used to retrieve more information from anisotropic samples [8]. In contrast to the aforementioned imaging techniques, which require highly dedicated hardware and software, CE-CT as discussed in this review is a more commonly used and affordable technique that allows to achieve a high sample volume to voxel size ratio.

Typically, two kinds of CAs are used in the field: perfusion CAs for *in vivo* imaging and CAs that bind to the tissues for *ex vivo* imaging. Here, we focus on the latter type of CAs, to be used for CE-CT-based quantitative “virtual 3D anatomical pathology.”

## 2. First Exploratory Studies on Contrast-Enhanced MicroCT

The very first reports focusing of the use of commercially available chemical compounds as CAs for CE-CT visualization of soft tissues go back to only about a decade ago. Johnson et al. [9], Litzlbauer et al. [10], and Ribi et al. [11] used osmium tetroxide (OsO_4_), a highly toxic stain, on mouse embryos, pig lungs, and honey bees respectively, to enable their virtual 3D anatomical analyses using CE-CT. Few years later, Metscher and his group went one step further by exhaustively investigating the staining potential of several commercially available chemical compounds as CE-CT CAs for different sample types [12, 13]. Their research about different sample fixations and staining protocols gave birth to the very first protocols for soft tissue CE-CT visualization. Metscher and coworkers demonstrated the use of very simple and versatile staining methods for quantitative volumetric imaging of animal soft tissues. They investigated which fixation method should be preferred for better imaging results, depending on the tissue type and the CA used. This research also confirmed that inorganic iodine (IKI) and phosphotungstic acid (PTA) could be used for a broad range of CE-CT applications. The main advantages of these two CAs are the simplicity of the sample preparation and their safer nature compared to OsO_4_. However, it should be noted that these studies only investigated the staining potential of the CAs for CE-CT of different soft tissues, without studying the potential destructiveness of the CAs themselves on the tissues or their binding mechanisms. From then on, more exploratory studies were performed to investigate the staining potential of new chemical compounds [14–16]. In 2013, Pauwels et al. reported an elaborate tissue-directed screening study of many different chemical compounds to be used as CE-CT CAs [17]. While this study reported various CAs for soft tissue imaging, many of them had a high osmolality compared to the biological samples, which could harm the samples due to tissue shrinkage. Moreover, the tissue-specific binding mechanisms still remained to be better understood. Other work focused on the validation of the method against established gold standards like (immuno-)histology [18].

In what follows, we will discuss several studies that paved the way for CE-CT-based virtual 3D anatomical pathology, and we will give educated opinions on the things to consider as well as on the remaining challenges and future perspectives. MicroCT has become over the years a standard imaging technique for 3D structural analysis of bone and mineralized tissues, and as a result, a majority of the studies that have reported on the use of CAs for CE-CT imaging have been performed with regards to the musculoskeletal system. Hence, this review will mainly focus on musculoskeletal tissues (Table 1) [3, 13, 15, 17–50].

## 3. Contrast-Enhanced MicroCT for Cartilage

The musculoskeletal system ensures locomotion, protection, and stability of the body. To fulfil its functions, this system is mainly composed of different tissues: long and flat bones, cartilage, muscles, tendons, ligaments, and other connective tissues. The visual assessment and structural analysis of these tissues is relevant for many different fields (i.e., regenerative medicine, drug development, early detection of pathologies, and developmental biology). Cartilage is one of the most studied musculoskeletal tissues with regard to CE-CT imaging. It is composed of a collagenous matrix that is rich in glycosaminoglycan (GAG) polysaccharide chains; because of these GAGs, cartilage tissue possesses a negative fixed charge density. As a consequence of its specific composition, CE-CT CAs for cartilage can be classified in three groups: anionic, cationic, and nonionic CAs.

For the first group of CAs, equilibrium partitioning of an anionic contrast agent *via* microCT (EPIC-microCT) has been described to study the morphology and composition of cartilage [19]. EPIC-microCT is a methodology in which a tissue sample is equilibrated in a solution of an anionic contrast agent. It relies on the inverse distribution of anionic contrast agents to the fixed negatively charged GAGs due to electrostatic repulsion, which thus negatively stains the tissue, making it appear darker than the surrounding tissues in the reconstructed images. Over the past years, several commercially available iodinated anionic CAs (e.g., iothalamate [26] (Cysto-Conray® II) and ioxaglate [19, 20, 21] (Hexabrix®)), which were originally developed for *in vivo* cardiovascular and urological imaging, have been studied for both qualitatively and quantitatively assessments of the cartilage morphology [19–22, 36, 51], but others have also been reported (Table 1) [26, 36, 52]. Apart from iodinated anionic CAs, other studies reported the use of gadolinium compounds (e.g., gadopentetate (Magnevist®)) as anionic CE-CT CA [42, 43]. The disadvantage of anionic CAs is the high concentrations required in order to obtain sufficient contrast-to-noise ratios [27]. Furthermore, the contrast and the overall quality of the images might no longer be sufficient for accurate quantification of the GAG content when its amount in the cartilage is too low (e.g., in engineered cartilage [38]).

To improve the potential of electrostatic interactions between the CA and the GAGs within the cartilage, iodinated cationic CAs have been developed, which attract rather than repulse GAGs, compared to anionic CAs. The synthesis and evaluation of such iodine-based, positively charged compounds for use as CE-CT CAs was first described by Joshi et al. [23]. Soon after, it was reported that a CA with a net positive charge of four (CA4+) was highly taken up in the cartilage tissue and, therefore, generated higher X-ray attenuation of the cartilage tissue at lower concentrations compared to the anionic CAs [24, 28, 29]. Moreover, the higher equilibrium concentration of CA4+ resulted in an increased sensitivity to the GAG content and, hence, a more specific imaging of the cartilage tissue and a more precise tissue differentiation [27, 30–32]. Furthermore, it has been demonstrated in *ex vivo* bovine osteochondral plugs, mouse tibia, and human cartilage that the increased X-ray attenuation (induced by CA4+) correlated to the equilibrium compressive modulus and the coefficient of friction [3, 29, 33]. One limitation of using CA4+ is, however, the considerably longer time required to reach diffusion equilibrium in comparison with anionic CAs [27, 29].

**Figure 1:**
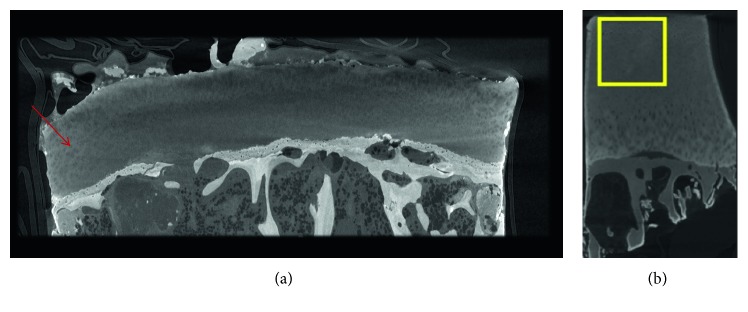
CE-CT of cartilage samples with POM staining. (a) Unpublished data: typical Hf-WD POM-based CE-CT cross section of an osteochondral sample of a human femoral head, clearly showing the individual chondrocytes within the articular cartilage layer, as indicated by the red arrow. (b) CE-CT cross section of an osteochondral sample stained with PTA (image from the study of Nieminen et al. [40]).

Besides ionic CAs, several nonionic CA exist. The main limitation of nonionic CAs (such as iopromide [36] (Ultravist®)) for cartilage imaging is the weak correlation between the GAG content and the X-ray attenuation. Therefore, most of the nonionic CAs are selected/developed to bind to the collagen in the cartilage matrix. PTA, for example, has been described for cartilage imaging because of its attractive interactions with collagen in an acidic environment (Figure 1(b)) [39–41]. Similarly to PTA, an Hafnium-based Wells-Dawson polyoxometalate (Hf-WD POM) was proven to bind to collagen [18] and has been used for CE-CT visualization of the cartilage tissue within human osteochondral samples, though within a neutral, nonacidic environment (Figure 1(a)) [18]. For both CAs, individual chondrocytes could be visualized within the cartilage matrix.

All things considered, CA4+ and related cationic CAs are highly promising for quantitative cartilage CE-CT imaging because of their high affinity and sensitivity to GAGs, making functional imaging of the cartilage tissue possible. When aiming to assess the structure of the cartilage tissue and the chondrocytes within, POM-based CAs have been proven to be highly potential. Research is still ongoing for optimization of all these different compounds.

## 4. Contrast-Enhanced MicroCT for the Bone Marrow Compartment

Bone organs are not only composed of mineralized tissues, but they also contain nonmineralized, soft tissues like bone marrow and adipose tissue, and vasculature. Thanks to the known binding power of osmium tetroxide (OsO_4_) to lipids, Scheller et al. reported the use of this CA for 3D CE-CT visualization of the bone marrow adipose tissue (BMAT) in long bones of mice [46, 47]. Using the reconstructed 3D images, they could quantify region-specific adiposity, which revealed the regulated and constitutive BMAT formation. They also unveiled the importance of BMAT in bone remodelling. Notwithstanding the widespread use of OsO_4_ as a staining dye for unsaturated lipids, it is highly toxic and has a limited tissue penetration capability [12]. Additionally, it requires a two-step scanning protocol: before and after bone decalcification.

A recent study within our group reported the simultaneous visualization of mineralized and soft structures within bones utilizing Hf-WD POM as CA [18]. Thanks to the combination of the hydrophobic behaviour of adipocytes and the binding of Hf-WD POM to the bone marrow tissue, Kerckhofs et al. were able to visualize the bone marrow adipocytes at the single cell level, using high resolution CE-CT scanning. This not only allowed to quantify the volume fraction of BMAT within the bone, but it also enabled the quantification of the adipocyte number/density and diameter. Additionally, the vascular network could be visualized and discriminated from the other tissues, allowing full 3D blood vessel network assessment (i.e., branching analysis and spatial distribution). Consequently, as Hf-POM-based CE-CT provides additional data to standard histomorphometry, with more spatial information, it could offer novel insights into the complex mechanisms of normal bone development and bone pathologies.

## 5. Contrast-Enhanced MicroCT for Muscle Tissue

A substantial amount of CE-CT studies have reported about the 3D visualization and structural assessment (muscle volume, fibre orientation, thickness, etc.) of muscle tissue. Based on this type of assessment, CE-CT not only allowed to better understand muscle function and development, but also to evaluate structural changes due to diseases. Similarly to magnetic resonance imaging, CE-CT of muscles could serve as input for computational modelling [53, 54], which could be valuable as complementary tool or validation for *in situ* loading studies, where displacements can be experimentally quantified to reverse engineer stress distributions [53]. Furthermore, a sufficiently high spatial image resolution should provide the feasibility of visualization, quantification, and modelling of individual muscle fibres.

For muscle tissue, Lugol's iodine (I_2_KI) and PTA are the most frequently reported CAs [13, 15, 17, 44, 45]. Although these two CAs provide very good staining of this type of tissues (and collagenous soft tissues in general), several studies reported shrinkage of the samples induced by the use of these CAs [48, 55, 56]. Tissue shrinkage is mainly caused by the low pH of PTA in solution and the high osmolality of I_2_KI. Shrinkage of the samples is a crucial effect that needs to be avoided or minimized when using CE-CT on soft tissues, as it changes the tissue morphology and integrity significantly, eliminating correct structural analysis and CE-CT image-based modelling. Consequently, analysis of the CE-CT data could be biased. Additionally, shrinkage damages the samples and the complementarity of CE-CT to other biochemical assays can thus be hampered. As a potential solution to this problem, Kerckhofs et al. recently reported that Hf-WD POM could provide similar muscle tissue staining as PTA, without inducing tissue shrinkage thanks to a physiological pH [18].

## 6. Contrast-Enhanced MicroCT for Tendons and Ligaments

In the musculoskeletal system, tendons and ligaments play an important role in motion and stability. They are mostly composed of collagen and have a highly fibrous structure. Several studies reported the use of iodine-based CAs (I_2_KI, Imeron300) or PTA for the 3D visualization of these collagenous tissues [13, 17, 48, 49, 50]. Rossetti et al. showed a proper example of using CE-CT as a complementary 3D imaging tool to micromechanical, compositional and proteomic methods in order to investigate the physiological functions of the bone-tendon insertion [50]. However, Balint et al. highlighted that I_2_KI staining allows 3D imaging of ligaments, but they did not succeed in obtaining sufficient contrast for the individual collagen fibres, and this CA invoked observable tissue shrinkage [48]. Sartori et al. have shown that PTA is able to reveal the individual fibres of the tendon, provided a sufficiently high spatial image and contrast resolution. Indeed, by scanning a demineralized PTA-stained Achilles tendon enthesis, they were able to visualize the collagen fibre bundles in the tendon. A known limitation of PTA is, however, again its acidic nature, inducing tissue shrinkage upon staining. When quantifying the structural parameters of the collagenous fibres in tendon or ligament tissue, this shrinkage will induce a significant bias. Therefore, Sartori et al. used phase contrast imaging rather than PTA staining for the structural quantification of the fibre thickness distribution and volume of the tendon [49]. Additionally, in case the biomechanics properties of these fibrous tissues were to be assessed by functional *in situ* testing (e.g., *in situ* loading of tendons within the microCT device, referred to as 4D microCT), the destructive character of these CAs would be an important limitation. Thus, there is still a pending need for a proper noninvasive CA for CE-CT assessment of fibrous tissues, like tendons and ligaments.

## 7. Remaining Challenges

Despite the substantial amount of advances in the field of CE-CT imaging for virtual 3D anatomical pathology and structural quantification of different biological tissues, some key challenges still remain to be overcome. One of the main limitations in the field is related to the limited sample size to voxel size ratio of the microCT devices themselves. This limitation can hamper the visualization of individual cells within an entire tissue. Using OsO_4_, an adipocyte-specific CA, Scheller et al. were able to image overall adiposity within murine long bones, but they could not perform single-cell analysis due to the limited spatial image resolution [18, 46]. In order to visualize and analyze individual adipocytes, Kerckhofs et al. focused on the analysis of the metaphysis of the bones only, which allowed to increase the spatial image resolution [18]. The same applies for the collagen fibres in tendons or ligaments; only with sufficiently high spatial image resolution, these features could be visualized. This shows the need of not only a suitable CA for good contrast, but also of sufficiently high spatial image resolution.

**Figure 2:**
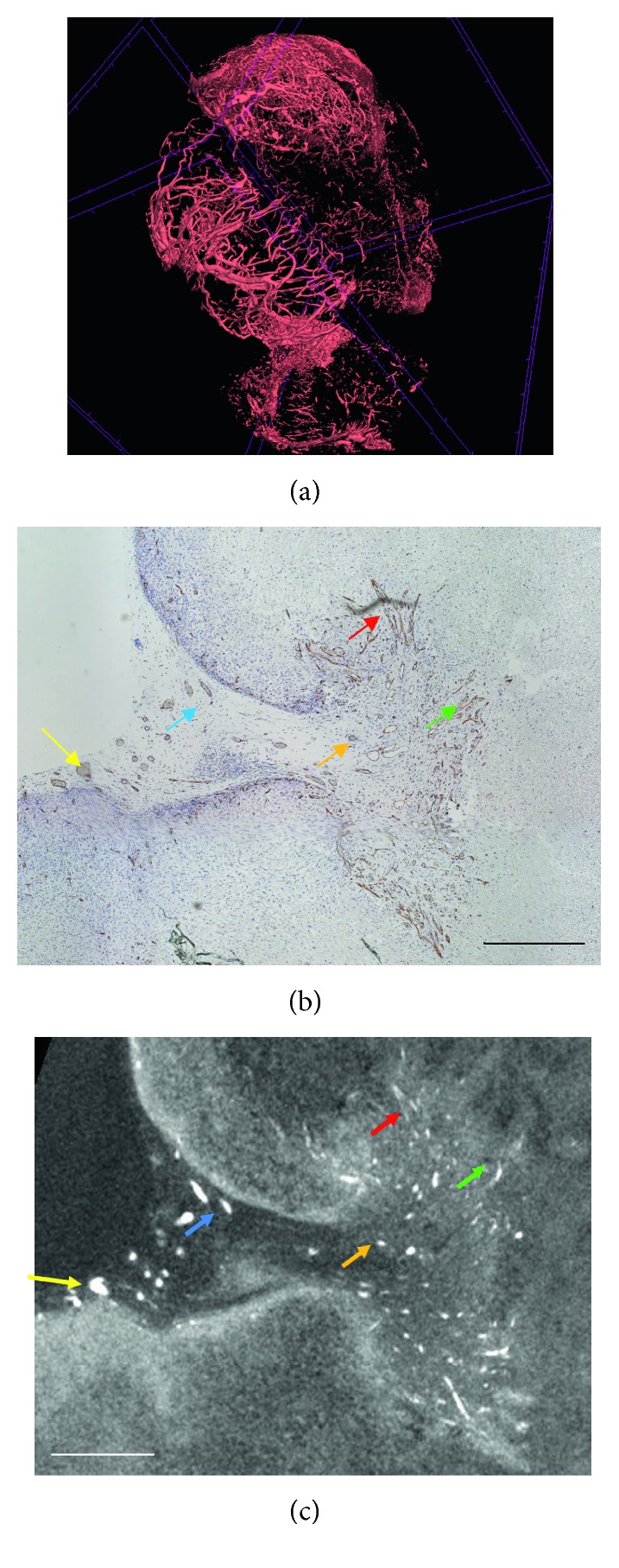
CE-CT images of vascularization in a tumour xenograft sample, adapted from Kerckhofs et al. [59]. (a) 3D rendering of the vasculature in a tumour xenograft, stained with Hf-WD POM; 3D scale bar = 100 *µ*m. (b) The CD31 stained section. (c) The corresponding CE-CT cross section through the tumour xenograft. The brown colour in the histological section indicates CD31-positive blood vessels. The white colour in the CE-CT image represents red blood cells in the blood vessels. The coloured arrows show corresponding blood vessels in both images. Scale bars = 100 *µ*m.

Another challenge in the field is the *ex vivo* CE-CT visualization and characterization of vascular networks. Indeed, once harvested, the vascularized networks within a sample are mostly opened up to the air causing possible leakage of the injected CA, which implies that the use of perfusion CAs, as for *in vivo* microCT, is challenging. Casting techniques have shown their potential [57, 58], but as most vascularized tissues contain not only large vessels but also small capillaries, in which the blood flow is inherently low, these techniques often do not reach capillaries, limiting accurate visualization and analysis of microvasculature. Hf-WD POM has been reported to allow visualization of the blood vessel network within the bone marrow by generating a negative stain of the blood vessels (i.e., staining of the surrounding hematopoietic tissue) when no red blood cells were present in the vessels [18]. Moreover, when this CA comes in contact with red blood cells, it will bind to them, allowing visualization of the blood within the vessel network of for example tumors (Figure 2) [59]. Although these results were validated against CD31 immuno-histological staining, more in-depth studies about the binding mechanisms between the Hf-WD POM and the red blood cells are still required.

Finally, although some cell- or tissue-specific CAs are reported in literature, the development of novel tissue-specific CAs still needs to be widely explored. The use of iron nanoparticles was reported for single cell visualization [60]. Because of the limited spatial resolution and restricted affinity of the nanoparticles to the cells, the minimum amount of detectable cells was 50 000 within muscle samples. Additionally, the nanoparticles needed to be administered inside the cells before implantation, questioning the potential cytotoxicity of the CA. Hence, future developments should focus on designing, synthesizing, and evaluating new high affinity, cell-specific CAs. One possible solution could be the development of CAs that are specific for antigens or proteins, i.e., immunological imaging. Literature regarding immunological imaging using CE-CT is, however, premature. To the best of our knowledge, only one group reported a metal-based immunodetection staining for whole body visualization of different genes [61]. A major disadvantage of the described enzyme product precipitation method was the appearance of background deposition of the precipitate and thus unspecific staining, and thus further development and optimization of immunological CE-CT CAs is required. 3D high-resolution immuno-specific CE-CT can be highly valuable as it could reveal relevant information about the spatial distribution of specific proteins or antigens within 3D heterogeneous tissues. Because of the high potential of these novel protein/antigen-specific CAs, we predict ground-breaking outcomes resulting from their use in different basic and preclinical research studies.

## 8. General Conclusions

Histologists have been working on stains for microscopy for more than 400 years since Anton Van Leeuwenhoek stained protozoans using saffron extract in the 1600s [62]. CE-CT is, however, still a very young field of research, only about a decade old, but it has a high potential within the field of biomedical research because of its minimally invasive character and its possibility to produce full 3D datasets that allow structural analysis of biological tissues. There is a strong need for this type of 3D imaging methodology, certainly for heterogeneous tissues, but it still requires further improvement, optimization, and validation. In this paper, we provided a nonexhaustive review of the current state-of-the art in the field of CE-CT imaging. While other groups reported CE-CT visualization of other types of tissues (nervous tissues [63], brain [11, 64], etc.), we focused here on musculoskeletal tissues, as for these tissues most of the progress in the field has been made.

Although there has been quite some progress in the applicability of CE-CT for the quantitative 3D assessment of biological tissues (i.e., quantitative virtual 3D anatomical pathology), the global acceptance of this innovative technology within the biomedical field is still a challenge. There are several limitations to overcome before quantitative virtual 3D anatomical pathology based on CE-CT will become a standardly used complementary technique to immunohistomorphometry. The destructiveness of the CAs and scanning protocols (1) should be reduced as much as possible to ensure that the tissue integrity is not harmed, and thus structural analysis is not biased, and that the technique is complementary to other assays (e.g., immunohistomorphometry) and functional testing such as 4D microCT. The specificity of the CAs (2) and their binding mechanisms to the tissues or cells should be more deeply understood to allow appropriate interpretation of the results, and novel CAs should be developed with well-known and tissue-/protein-/antigen-specific binding capacities. Standard operating procedures (3) for sample preparation, staining, and acquisition should be developed to increase the comparability, the ability to share, and the reproducibility of the results. Indeed, optimization of the staining parameters such as concentration and staining time (4) for every tissue type is of substantial importance to maximize CA diffusion, and thus staining, and minimize potential tissue shrinkage. Additionally, the scanning parameters (5) ultimately have a nonnegligible influence on the qualitative and quantitative assessment of the tissues. Overcoming these limitations will be crucial to move the field of CE-CT imaging one step further towards a standard accepted tool for quantitative virtual 3D anatomical pathology.

## Figures and Tables

**Table 1:** Reported CE-CT contrast agents used for musculoskeletal tissues.

Tissue	CAs and references	Remarks and added value of CE-CT
Cartilage	(i) Anionic iodinated CA (1) Ioxaglate/Hexabrix® [19–25] (2) Iothalamate/Cysto-Conray® II [23, 26] (3) Gadopentetate/Magnevist [24] (ii) Cationic iodinated CA (1) CA4+ [3, 23, 24, 27–35] (2) CA1+ [23] (3) CA2+ [23] (iii) Nonionic iodinated CA (1) Iopromide/Ultravist® [36] (2) Iodixanol/Visipaque® [37] (iv) PTA [38–41] (v) Gadopentetate dimeglumine/Magnevist® [24, 42, 43] (vi) Gd^3+^ [42] (vii) Gadoteridol [35, 42] (viii) Hf-WD POM [18] (Figure 1(a))	Electrostatic interactions between anionic or cationic CAs and the GAGs in the cartilage enable quantification of GAG content POM CAs bind to collagen, enabling structural analysis of the cartilage tissue and, in case of sufficiently high spatial resolution, imaging of individual chondrocytes

Bone marrow compartment	Hf-WD POM [18]	Enables simultaneous visualization and structural quantification of adipocytes, vasculature, and mineralized tissues

Muscle	PTA [13, 15, 17] I_2_KI [13, 44, 45] Hf-WD POM [18] PMA [17] HgCl_2_ [17] Na_2_WO_4_ [17] (NH_4_)_2_MoO_4_ [17]	PTA, PMA, I_2_KI, HgCl_2_, Na_2_WO_4_, and (NH_4_)_2_MoO_4_ have high osmolality compared to biological tissues, thus inducing tissue shrinkage

Bone marrow adiposity	OsO_4_ [46, 47]	OsO_4_ is highly toxic and requires a two-step scanning protocol

Tendons and ligaments	PTA [13, 17, 48, 49] I_2_KI [49] Imeron300 [50] PMA [17, 48] (NH_4_)_2_MoO_4_ Ba(ClO_3_)_2_ [17] HgCl_2_ [17] Na_2_WO_4_ [17] BaCl_2_ [17]	Same remarks as for muscle tissue for PTA, I_2_KI, PMA, HgCl_2_, Na_2_WO_4_, (NH_4_)_2_MoO_4_, BaCl_2_, and Ba(ClO_3_)_2_
